# Controlled assembly of artificial 2D materials based on the transfer of oxo-functionalized graphene†

**DOI:** 10.1039/c9na00594c

**Published:** 2019-11-19

**Authors:** Marleen Hußmann, Benjamin Weintrub, Patrick Feicht, Gregor Germer, Jan N. Kirchhof, Kirill I. Bolotin, Siegfried Eigler

**Affiliations:** Institute of Chemistry and Biochemistry, Freie Universität Berlin Takustraße 3 14195 Berlin Germany siegfried.eigler@fu-berlin.de; Institute of Physics, Freie Universität Berlin Arnimallee 14 14195 Berlin Germany

## Abstract

Functionalized 2D materials have unique properties, but are currently not used for the assembly of van der Waals heterostructures. Here, we present the controlled transfer of artificially synthesized, polar and highly transparent oxo-functionalized graphene, which can decouple graphene layers.

## Abstract 2

A method is described to transfer and integrate transparent and polar 2D materials into heterostructures.

## Graphical abstract

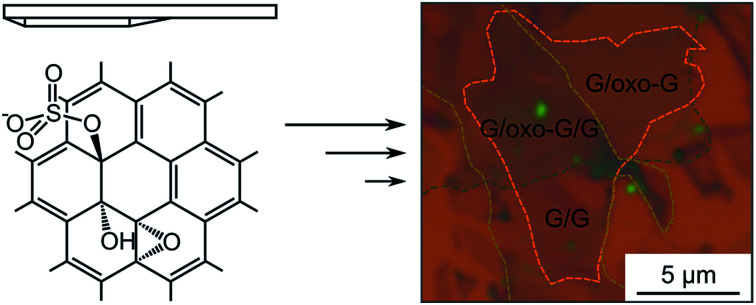

The fast-growing family of two-dimensional materials includes diverse members ranging in properties from insulators (*e.g.* h-BN and graphene oxide)^1^ and semiconductors (MoS_2_, WS_2_, and PtSe_2_)^2–4^ to semimetals (graphene and TiS_2_)^5,6^ and superconductors (NbSe_2_).^7^ Recently, it became possible to assemble dissimilar 2D materials into heterostructures. Such heterostructures can exhibit properties not seen in individual building blocks such as exotic superconductivity, modified band structures, and superlattice effects.^8–12^

To date, single layers of various 2D materials of the highest quality have been produced by mechanical exfoliation of their multi-layered crystals. In the case of graphene, the production can also be carried out by wet-chemical intercalation and oxidation of graphite.^13–15^ The thus obtained graphite oxide can be gently delaminated *via* osmotic swelling yielding graphene oxide flakes with a typical size of up to 100 μm.^16–18^ Graphene oxide belongs to a class of materials with variable sizes of sp^2^-domains isolated by defects (*e.g.* functionalization and vacancy defects).^19^ In particular, we here use our recently introduced oxo-functionalized graphene (oxo-G), which is a graphene oxide derivative with a very low defect concentration.^14,20,21^ Its density of in-plane lattice defects as well as the surface chemistry are well controlled.^20,22–24^ Oxo-G possesses hydroxyl, epoxy and organosulfate groups with hydronium counter ions as surface functional groups, resulting in a highly polar and protic surface with a negative zeta-potential.^17,25^ After reduction of oxo-G to a certain quality of graphene (red-oxo-G) only vacancy and hole defects remain. The presence of defects leads to the characteristic photoluminescence (PL) spectrum that can be used as a fingerprint of the material in a heterostructure.^26–28^ In this field, a graphene/ozone-treated graphene heterostructure was already prepared as a transparent conductive electrode for an organic light emitting diode.^29^ However, no controlled transfer of chemically modified graphene was demonstrated (Fig. 1).

**Fig. 1:**
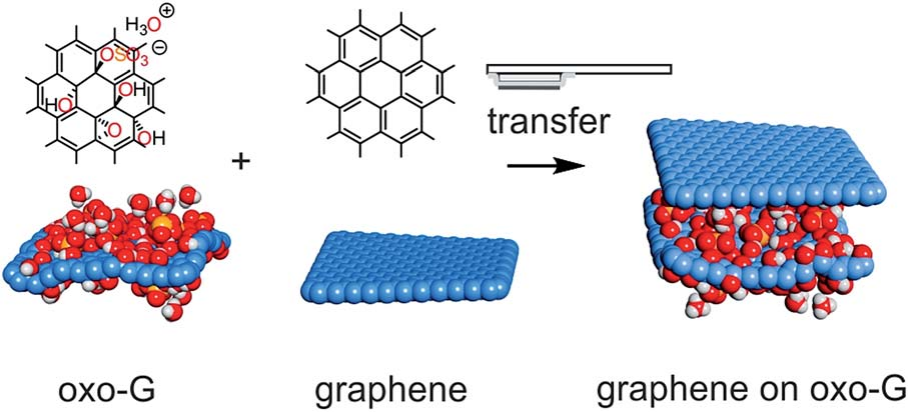
Illustration of the transfer of flakes: a flake of graphene (red-oxo-G) is transferred by a stamp on top of a flake of oxo-G to form a heterostructure of graphene (red-oxo-G) on oxo-G. Transfer of oxo-G on graphene is also possible.

In recent years the chemical modification of 2D materials such as functionalized MoS_2_,^30^ graphene,^31^ phosphorene,^32,33^ and antimonene^34,35^ have been investigated by various working groups. Although flakes of functionalized 2D materials were obtained, the controlled transfer of those flakes is not yet considered for the assembly of heterostructures. In particular oxo-G flakes available in large quantities were not yet transferred in a controlled way, although there are transfer techniques for exfoliated monolayers of *e.g.* graphene, MoS_2_, or other transition dichalcogenides.^36–40^ Flakes of oxo-G were used to improve perovskite solar cells,^41^ and organosulfate groups in oxo-G were exploited as templates for incorporating molecules, forming hybrid materials suitable for the fabrication of low-voltage memory devices.^42^ However, to establish the fabrication of functional heterosystems, which include oxo-G or oxo-G hybrid materials the controlled transfer of highly transparent and polar flakes of graphene oxide and oxo-G, respectively, must be established.

Here, we systematically investigate the transfer of flakes of oxo-G to make vertical heterostructures, including a structure of graphene/oxo-G/graphene (G/oxo-G/G).

First, we tested to make oxo-G/oxo-G and red-oxo-G/oxo-G structures by using the Langmuir–Blodgett technique. However, the approach is random and thus limited in terms of controlling the appropriate size, shape and quality of flakes used for the assembly. Accordingly, the assembly of specific stacks of oxo-G derivatives using this method is highly unlikely. The details of this approach are summarized in the ESI (Fig. S1†).

For the controlled assembly of specific heterostructures, flakes of oxo-G were first deposited by the LB technique on Si/300 nm SiO_2_ wafers. The size and quality of flakes are subsequently probed by optical microscopy, atomic-force microscopy (AFM) or Raman spectroscopy. Flakes of interest are selected, as shown in Fig. 2A and B. For the transfer, a polydimethylsiloxane (PDMS) stamp is used with a sacrificial polymer layer consisting of poly(methylmethacrylate-*co-n*-butylmethacrylate) (short term: Elvacite 2550), as reported in previous work.^36^ A schematic representation of the transfer slide is shown in Fig. 3B. The stamp is brought into direct contact with the desired flake, controlled by optical microscopy. The pick-up of oxo-G flakes is facilitated with additional capillary force resulting from applying water vapor as described in the experimental section.^37^ The release is achieved by heating the substrate to 120 °C melting the Elvacite polymer (setup is described in the ESI and Fig. S2†). Afterwards, the substrate is cooled to 90 °C to ensure the integrity of the flake while removing the transfer slide. The successful transfer of the oxo-G flake is shown in Fig. 2C on top of the oxo-G flake of Fig. 2A.

**Fig. 2:**
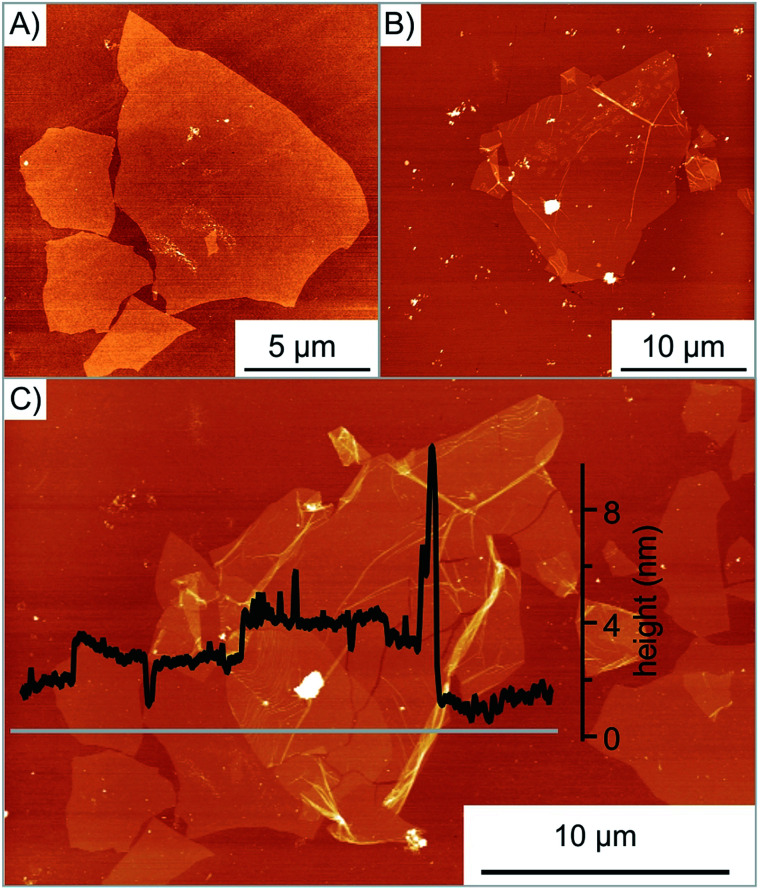
Transfer of oxo-G on oxo-G. (A) AFM image of the bottom oxo-G flake before transfer. (B) AFM image of the oxo-G flake to be transferred. (C) AFM image of the successful transfer; inset: height profile along the grey line.

**Fig. 3:**
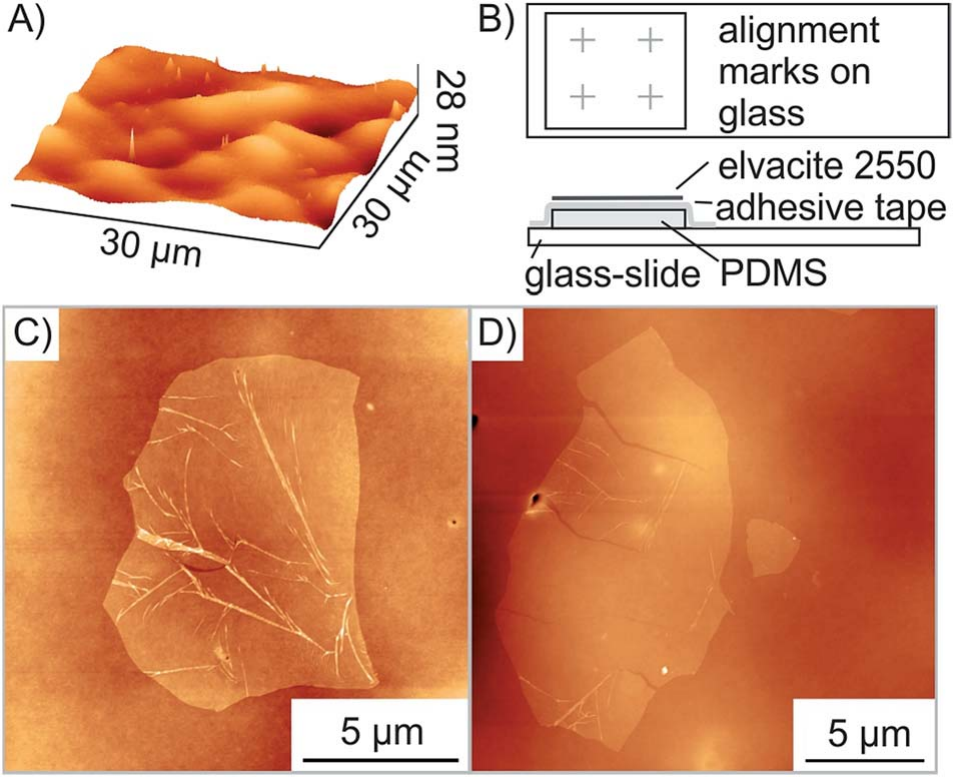
Roughness of the surface leads to wrinkles and cracks respectively in the flake. (A) AFM image of the Elvacite surface prepared by drop-casting with a mean roughness *R*_a_ of 2.0 nm. In comparison, the *R*_a_ of Elvacite deposited by spin-coating is 3.8 nm which hinders the pick-up. (B) Schematic assembly of the transfer-stamp with alignment marks (grey crosses). (C) AFM image after transfer of a flake on Elvacite located in a valley showing wrinkles. (D) AFM image after transfer of another flake on Elvacite located on a hill displaying cracks.

While performing these transfer processes several obstacles emerged. For example, Raman mapping associated with laser-induced heating, conducted on the desired oxo-G flake before transfer, may inhibit the pick-up.

We propose that water molecules underneath the flake may determine the adhesion to the SiO_2_ surface. This assumption is supported by the observation that films of oxo-G flakes deposited by the LB-technique on the SiO_2_ substrate do sometimes not adhere to the SiO_2_ substrate, which is very likely due to a film of water between the substrate and oxo-G flake.^37,43,44^

As described by Zomer *et al.* flakes of graphene and h-BN can be transferred using a polymer coated adhesive tape on a PDMS stamp placed on a glass slide.^36^ The oxo-G flakes and red-oxo-G flakes are barely visible on Elvacite using optical microscopy (see Fig. S3A†). When looking through the media (glass, PDMS, tape, Elvacite) of the transfer slide, the flakes are not visible during the transfer process. Locating flakes is however possible using impurities on the Elvacite polymer, such as few-layers or dust, which act as reference points. Nevertheless, these dust particles are more often not gathered along with the desired flake, losing the reference points and thereby also the flake. For this reason, we used standard electron-beam lithography to create alignment marks using 3 nm Cr/20 nm Au on the glass slide substrate (Fig. 3B, see Fig. S3B†). Furthermore, we surprisingly recognized that a PDMS thickness of less than 0.5 mm increases the visibility of oxo-G flakes and thus facilitates the location and positioning of flakes.

As depicted in Fig. 2C, the transfer process damaged the transferred flake. The height profile shows both the successful overlap of two flakes and emerged wrinkles as well as cracks. Moreover, multiple folding is observed with a height of 10 nm. In order to determine the cause of additional wrinkles and cracks, the surface roughness of the exposed Elvacite film was evaluated by AFM before and after pick-up of flakes. Fig. 3A shows that the Elvacite surface is composed of hills and valleys roughly 15 nm peak-to-peak, and flakes may be placed in valleys with the tendency to form wrinkles (Fig. 3C) or on hills leading to cracks (Fig. 3D). Regarding the roughness, we observe that a roughness of 2.0 nm guaranties an extensive pick-up of flakes while a roughness of 3.8 nm is not sufficient to pick up flakes reliably. The low roughness of Elvacite can be achieved (see Fig. S4†) by drop-casting a solution of Elvacite followed by inclining the substrate and letting the solution run down. In contrast a standard spin-coating process leads to an insufficiently high roughness (see ESI†).

With the developed method we prepared artificial stacks of 2D materials, in particular oxo-G on oxo-G and red-oxo-G on oxo-G and also mechanically exfoliated graphene on oxo-G. The results of the transfer approach are presented in Fig. 4, and the different heterostructures are characterized by Raman microscopy. In Fig. 4B the Raman map of the oxo-G on oxo-G transfer (Fig. 4A) is shown. The overlap is accompanied by the increase of the intensity of the D peak, which is also demonstrated in the AFM image of Fig. 4C and by the average spectra of the bottom oxo-G (red line), the top one (blue line) and the overlap (green line). Herein the intensities (integrated area) of the peaks in the single spectra contribute in first approximation additively to the spectrum of the overlap (see Fig. S5†).

**Fig. 4:**
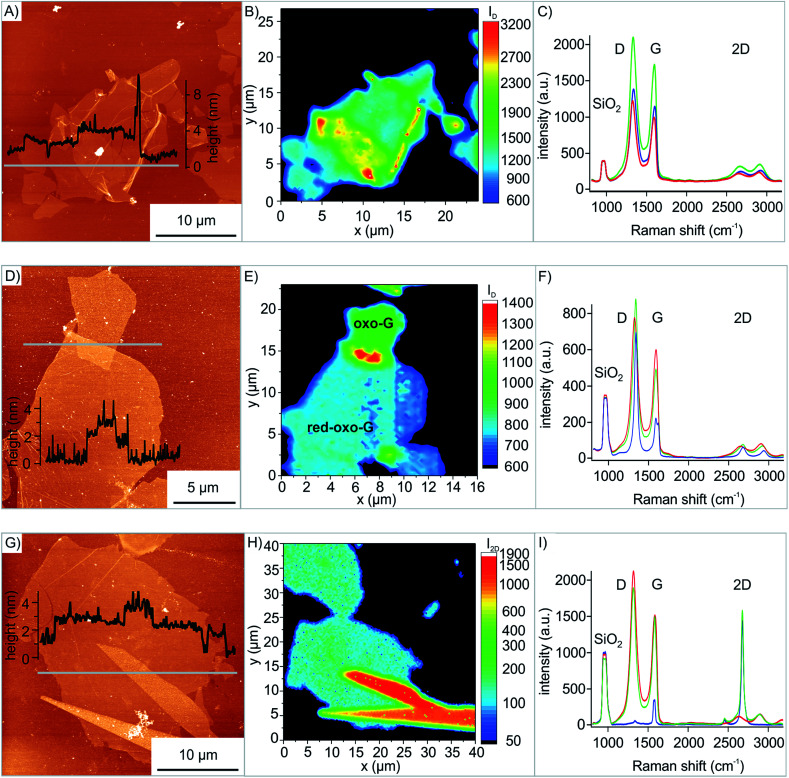
AFM and Raman measurements of three different transfers. (A) AFM image of the transfer of oxo-G on oxo-G; inset: height profile along the grey line. (B) Corresponding Raman map. (C) Average spectra of the top oxo-G (blue), bottom oxo-G (red) and the overlap (green). (D) AFM image of the transfer of red-oxo-G on oxo-G; inset: height profile along the grey line. (E) Corresponding Raman map. (F) Average spectra of red-oxo-G (blue), oxo-G (red) and the overlap (green). (G) AFM image of the transfer of mechanically exfoliated graphene on oxo-G; inset: height profile along the grey line. (H) Corresponding Raman map. (I) Average spectra of graphene (blue), oxo-G (red) and the overlap (green).

Next, transferring of red-oxo-G was conducted. While a partial overlap of oxo-G on oxo-G and on red-oxo-G respectively is possible with LB, it is not for red-oxo-G on oxo-G because overlapping oxo-G flakes would be reduced by reducing agents. The transfer was performed in the same way as described earlier for oxo-G. The result is shown in the AFM image of Fig. 4D. The height profile of the AFM measurement indicates the overlapping area with a height of approximately 3 nm compared to red-oxo-G with about 1 nm height and oxo-G with a height of about 2 nm. Furthermore, the Raman map of the intensity of the D band is shown in Fig. 4E, in which the more intense, red coloured scale corresponds to the overlap.

The average spectra of the overlapping red-oxo-G/oxo-G region resemble the individual spectra of the red-oxo-G flake and the oxo-G flake. In addition, the transfer of mechanically exfoliated graphene onto oxo-G (oxo-G/G) was conducted to prove that other 2D materials, here in particular graphene can be transferred onto the flakes of oxo-G. In Fig. 4G the AFM image as well as the height profile shows the overlapping area of flakes with some glue residue from the tape on the graphene. The corresponding Raman map in Fig. 4H depicts the intensity of the 2D peak, used to visualize the different materials stacked on top of each other.

The average spectra in Fig. 4I, generated from single spectra obtained by Raman mapping, exhibit a sharp 2D peak for graphene and virtually no defect-induced D peak, while oxo-G is highly functionalized. Comparing the 2D peaks, it is found that the overlap is the sum of the graphene and the oxo-G intensity (see Fig. S5C†).

Finally, we demonstrate that oxo-G deposited between two layers of CVD graphene (G/oxo-G/G) leads to decoupling. The structure was then heated to 100 °C so that impurities could diffuse out more easily and the structure could relax. The 2D peak measured (Fig. 5E) is the sum of individual 2D peaks, instead of a broadened 2D peak expected for bilayer graphene.^45^ However, we propose that a random orientation of the graphene layers prevents perfect stacking as observed for bilayer graphene. Also impurities on the CVD graphene layer, as seen in Fig. 5C and in the AFM image in Fig. S6,† may influence properties. However, Jeon *et al.* determined the critical distance for decoupling graphene layers to be 1.66 nm maximum depending on their orientation.^45^ It is clearly seen in the height profile of the AFM image (Fig. S6†) that oxo-G spaces the graphene layers according to its height of 2 nm. Thermal processing (tp) of that structure leads to the disproportionation of oxo-G into a ruptured carbon framework and CO_2_.^22^ As depicted in Fig. S7† the Raman 2D peak redshifts by 11 cm^−1^ from 2684 cm^−1^ for G/oxo-G/G to 2695 cm^−1^ for G/tp-oxo-G/G indicating the change of doping from p to n for the trilayer structure.^46^

**Fig. 5:**
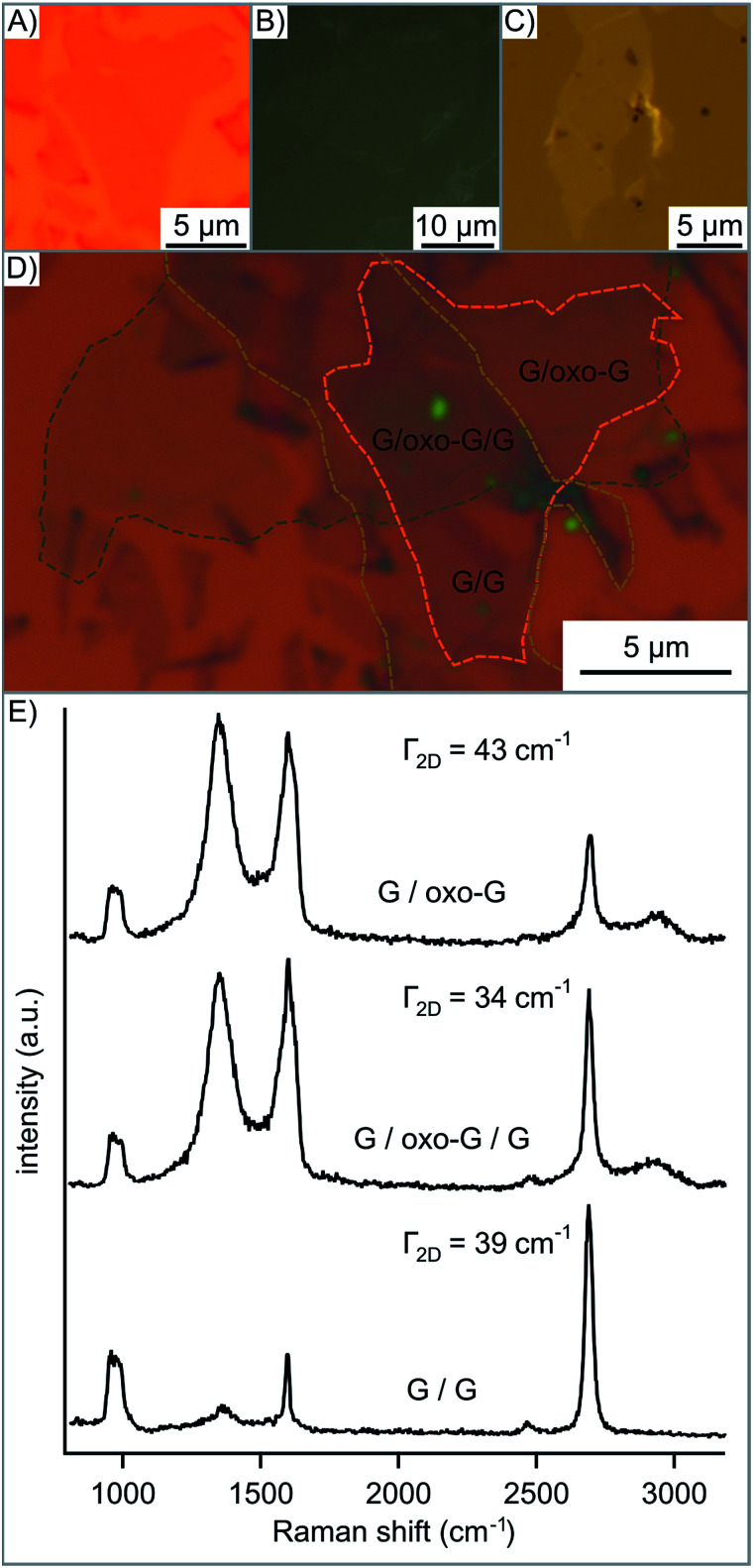
Heterostructure of graphene/oxo-G/graphene (G/oxo-G/G). (A) 1^st^ layer CVD graphene flake. (B) 2^nd^ layer oxo-G on the transfer slide. (C) 3^rd^ layer CVD graphene flake on the transfer slide. Pictures of (B) and (C) are mirrored so that each layer can be identified more simply within the complete structure in (D). (E) Raman spectra in the area of G/oxo-G, G/oxo-G/G and G/G, respectively.

## Conclusion

We introduced the controlled transfer of highly transparent oxo-G and demonstrated the specific assembly of oxo-G/oxo-G, oxo-G/red-oxo-G, oxo-G/G and also G/oxo-G/G, which identifies oxo-G as an interfering layer. We solved inherent problems of transferring oxo-functionalized graphene derivatives, which are barely visible and may crack or fold. We propose that a layer of water between the surface and oxo-G derivatives allows successful transfer. Moreover, by preparing a marker labelled glass substrate for the transfer stamp the precise orientation of transferred flakes becomes possible. The method is compatible also for transferring graphene and very likely other 2D materials, such as h-BN, TMDs or functionalized 2D materials in general. In future research the developed technique can be used to incorporate functionalized 2D materials into heterostructures to fabricate optoelectronic devices. In addition, the influence of defects in graphene on the properties of heterostructures can be studied. In general, with the presented method, novel 2D heterostructures with unexplored properties become accessible.

## Conflicts of interest

There are no conflicts to declare.

## Supplementary Material

NA-002-C9NA00594C-s001
