# Supplementary material for: Controlled assembly of artificial 2D materials based on the transfer of oxo-functionalized graphene
Source: Nanoscale Adv. 2019 Nov 19;2(1):176–81. doi: 10.1039/c9na00594c (PMC9417582; doi:10.1039/c9na00594c)
Supplement: NA-002-C9NA00594C-s001 [file NA-002-C9NA00594C-s001.pdf]

Electronic Supplementary Material (ESI) for Nanoscale Advances.
This journal is © The Royal Society of Chemistry 2019

Controlled assembly of artificial 2D materials based on the transfer of oxo-functionalized graphene

Marleen Hußmann,[a] Benjamin Weintrub,[b] Patrick Feicht,[a] Gregor Germer,[a] Kirill Bolotin,[b] Siegfried Eigler[a]*

[a] Institute of Chemistry and Pharmacy, Freie Universität Berlin, Takustrasse 3, 14195, Berlin, Germany, e-mail: siegfried.eigler@fu-berlin.de
[b] Department of Physics, Freie Universität Berlin, Arnimallee 14, 14195 Berlin, Germany

Materials and methods.
The chemicals were purchased and used as received, if not otherwise stated. Methanol (VWR, distilled before use), doubly distilled water (Carl Roth), hydrogen peroxide (30%, Carl Roth), $KMnO_4$ (Grüssing GmbH), H_2SO_4 (Sigma Aldrich), graphite (Asbury Carbon, grade 3061), trifluoroacetic acid (Merck) and hydrogen iodide (Acros Organics), Elvacite 2550 copolymer (Lucite International), Sylgard 184 (Dow Corning), methyl isobutyl ketone (Carl Roth GmbH), PMMA 950 A11 and PMMA 495 A11 (MicroChem).
Kibron MicroTroughXS was used for Langmuir-Blodgett procedure. Raman spectra were recorded with Horiba Explorer at 532 nm excitation wavelength (70 mW @ 100%). XYZ adjustable table was used for scanning Raman microscopy. A 100x magnification objective and a diffraction grating with 1200 lines/mm were used. The polarization of the laser was horizontal, the polarization of the detector horizontal, slit and hole were set to 500 and 200, respectively. The statistical dataset consists of at least 700 data points. Every data point has an acquisition time of 4 s and one accumulation.
AFM images were recorded in tapping mode using Nano Wizard 4 from JPK. Transfer stage was home built with temperature control unit and XY micromanipulators and combined with an optical microscope.
Optical microscope (Nikon Eclipse LV100ND equipped with a Nikon TU Plan ELWD 20x/0.40 objective.
Optical microscope (Nikon LV150N with DIC equipped with CFU TU Plan Fluor objectives 50x or 100x)
Spin-coater Laurell Technologies Corporation, WS-650MZ-23NPPB at 3000 U/min, 45 sec for Elvacite (concentration 150 g/L).
E-beam lithography, Raith Pioneer TWO;
Metal evaporator (Kurt J. Lesker NANO 36 with a FTC 2800 controller)

Preparation of flakes (oxo-G and red-oxo-G)

Oxo-G was synthesized according to the published procedure.[1]

The Langmuir-Blodgett process,[2] was used for coating the silicon wafers with 300 nm thermally grown SiO_2 surface. A dispersion of oxo-G in water/methanol (1:1 ratio) was added drop by drop on the water surface so that a constant surface tension of 1.3 $\frac{mN}{m}$ was achieved. The new surface tension was then set as the zero point. The barriers were closed until a surface tension of 3.0 $\frac{mN}{m}$ was reached. For the coating oxo-G on red-oxo-G, the Langmuir-Blodgett process was repeated after reduction.

Reduced oxo-G was prepared by reduction of oxo-G by trifluoroacetic acid and hydrogen iodide at 80 °C for 5 min. Afterwards the wafer was annealed in air at 140 °C for 5 min. In addition, the wafer can be rinsed with water.

Assembly of oxo-G/oxo-G and red-oxo-G/oxo-G structures by Langmuir-Blodgett technique.

Based on Langmuir-Blodgett-technique (LB), individual flakes of oxo-G in dispersion can be deposited homogeneously on Si/ 300 nm SiO_2 wafers. Bi- or few layers of homo- and heterostructures can be generated by repeated deposition application. For example, a first layer of oxo-G flakes may be chemically reduced before deposition of a second layer of oxo-G (**Fig. S1B**). However, the LB approach to obtain heterostructures is random and therefore limited in precision. Moreover, due to the heterogeneity of the material, it is very unlikely that a flake with intended structure will be deposited to the desired position. **Fig. S1A** shows the results of random assembly of flakes of oxo-G on oxo-G and **Fig. S1B** of oxo-G on red-oxo-G, respectively. Nevertheless, it is impossible either to predict or to control the overlapping region or to select flakes with a specific size and quality. The quality of flakes of oxo-G as well as red-oxo-G can be probed by statistical Raman spectroscopy.[3] The density of defects was determined interpreting the intensities and ratios of the D, G and 2D bands, according to the model of Lucchese and Cançado.[4, 5] The heterogeneity of materials, in particular graphene, can be visualized by plots of I_D/I_G ratio *vs.* full-width at half-maximum of the 2D band (Γ_{2D}, **Fig. S1C**).[3] Flakes of oxo-G (**Fig. S1C**, red) have a high degree of functionalization of the carbon lattice and therefore I_D/I_G ratio about 1 and Γ_{2D} > 150 cm^{-1}. Flakes of red-oxo-G (**Fig. S1C**, blue) show in average an I_D/I_G ratio of 3 with Γ_{2D} about 70 cm^{-1}. The assembled layers of oxo-G on red-oxo-G is shifted to an I_D/I_G ratio of 1.4 and Γ_{2D} between 50 and 300 cm^{-1}.

We conclude that random deposition is limited in terms of controlling the appropriate size, shape and quality of flakes used for the assembly. Accordingly, the assembly of specific stacks containing oxo-G derivatives is highly unlikely by this method.

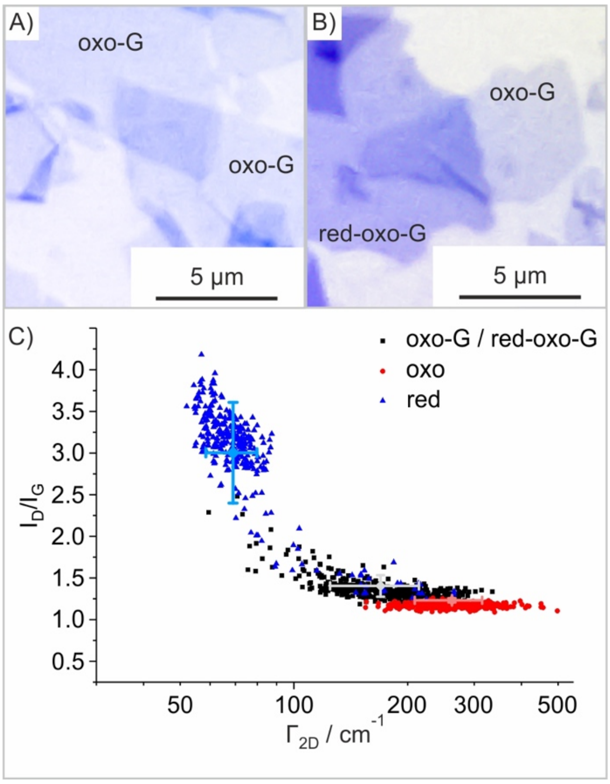

Fig. S1. Overlapping flakes prepared by repetitive Langmuir-Blodgett technique. Optical microscope image in differential interference contrast mode of flakes of A) oxo-G on oxo-G and B) oxo-G on red-oxo-G. C) Statistical Raman spectroscopy of films of flakes of oxo-G, red-oxo-G and oxo-G flakes deposited onto red-oxo-G flakes.

Fabrication of transfer slide with alignment marks and PDMS stamp

PDMS precursor (Dow Corning Sylgard 184) is mixed in a 20:1 mass ratio of oligomeric base to curing agent, degassed and poured into a polypropylene Petri dish. After prehardening for 20 h at room temperature, the samples are post-treated for 4 h at 80 °C. Then 2x2 cm^2 stamps are cut out.

The transfer method and therefore the transfer slide is based on the description of Zomer *et al.*.[6] However, the setup of the transfer slide is slightly changed. The used transfer slide is built up by a glass slide with a 1 mm thick PDMS stamp. Scotch tape is fixed tightly on top. A poly(methylmethacrylate-co-*n*-butylmethacrylate) copolymer (Elvacite 2550) dissolved in methyl isobutyl ketone (150 g/L) is dropped onto the slide and then the slide was tilted to achieve a thin uniform coating with a plane surface. The other transfer slide was spin coated for 45 s at 3000 rpm.

Transfer method for flakes of oxo-G and red-oxo-G

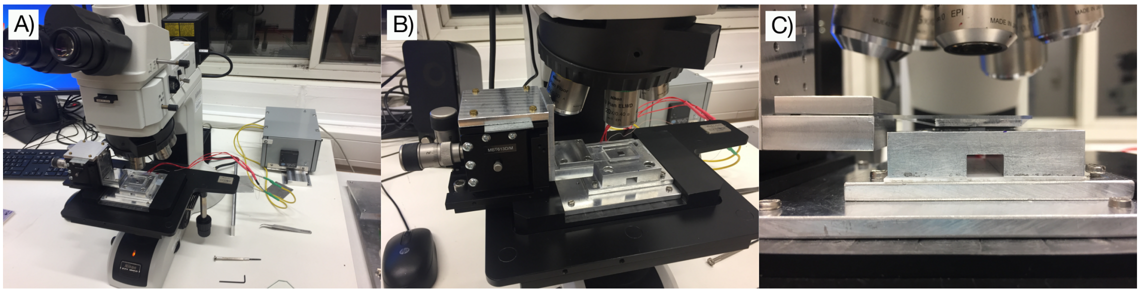

Fig. S2. Transfer station. A) Microscope with heating station on the right. B) Micromanipulator holding the transfer slide over the heating plate. C) Side view of the transfer slide and heating plate.

The transfer for oxo-G and red-oxo-G were performed with an optical microscope (see **Fig. S2**), a self-built heating plate with a micromanipulator and a thermoelement to control temperature, similar as describe in the literature[7] as follows: First, the to be transferred flake has to be located on the wafer. Then, the transfer slide is put into the micromanipulator upside down worsen the visibility. In order to pick up flakes from the wafer we take advantage of the capillary force as proposed by Ma *et al.* by using a steam treatment of the surfaces right before the pick-up.[8] Bringing the transfer slide about 1 mm close to the vicinity of the wafer, steam is blown between transfer slide and wafer, generating a highly dispersed covering area of water bubbles on the wafer. The steam can be prepared by different ways, such as passing air over a warm water supply. As long as the liquid is present, contact to the wafer is made and pressed until the Elvacite show a yellow-orange color. The position of the flake on the transfer slide is captured by refocusing to the alignment marks on the glass slide of the transfer slide (**Fig. S3B**). The pick up is performed very slowly depending on the observation of optical changes during the pick up. Removing the transfer slide out of the micromanipulator and looking upside at the picked up flake, it is vertically mirrored and better visible lying on a black background (**Fig. S3A**).

The release process comprise the melting of Elvacite. First, a destination flake has to be found. In the case of oxo-G, the location of the flake has to be visualized by means of some marks of the computer program of the microscope. Then, the transfer slide is put again into the micromanipulator. The location of the flake on the transfer slide, easily identified due to the alignment marks, has to be matched roughly in an area of 4 mm^2 with the position of the oxo-G on the wafer. Otherwise the wafer has to be realigned. Afterwards, the wafer is fixed with double sited tape on the heating plate. Following, the fine alignment takes place, bringing the transfer slide closer to the wafer, always checking their overlap. When contact is made somewhere on the wafer, seen at the rainbow like color changes in the Elvacite, further movement should be avoided to not rip the flakes. Then the heating plate was heated up until 120 °C and afterwards turned off. A comprehensive contact is generated, seen at a now

homogeneously color. Waiting until the temperature of the heating plate is 90 °C, the release of the flake is performed by lifting the transfer slide slowly and steady of about 20 s/mm. The polymer is subsequently washed away by putting the wafer in acetone for one day and rinsed with douple destillated water.

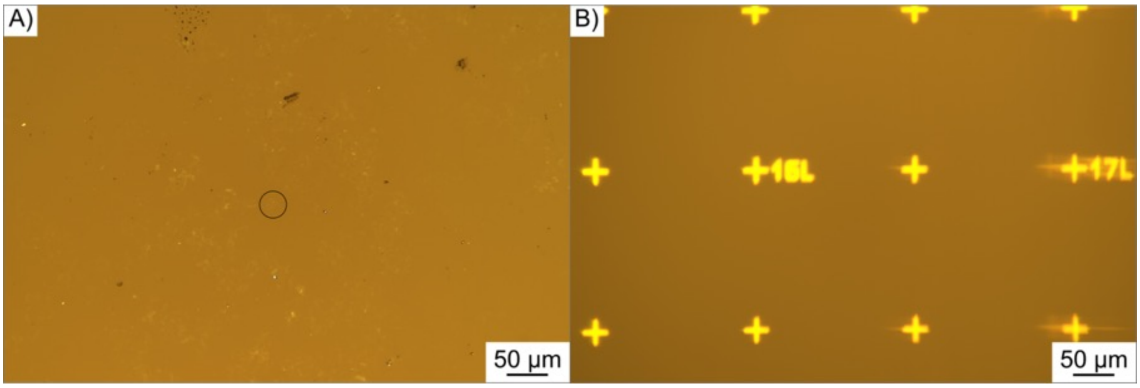

Fig. S3. Optical images of the transfer slide with focus on A) the oxo-G flakes on Elvacite, encircled the transferred oxo-G; also dust particles can be seen. B) Focus on alignment marks.

Roughness of Elvacite

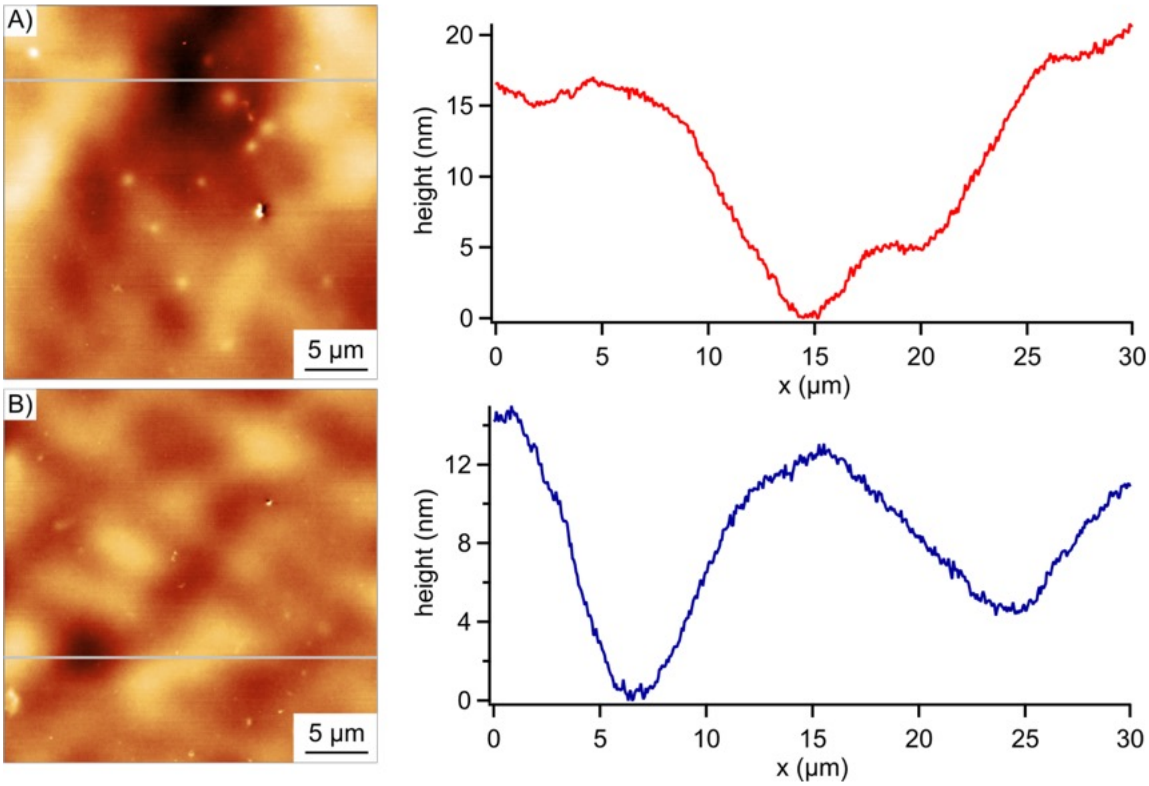

Fig. S4. Roughness of Elvacite A) AFM of spin-coated Elvacite, right: height profile along grey line, revealing a hill-to-hill distance of about 15 µm, B) AFM of drop-casted Elvacite, right: height profile along grey line; revealing a shorter hill-to-hill distance of 10 µm and a shallower valley.

Comparison of overlapped and calculated Raman spectra

Overlapped Raman spectra are approximately the sum of their single spectra (see **Fig. S5**). However, the Raman spectra of the heterostructure of red-oxo-G on oxo-G fits better with its arithmetic average.

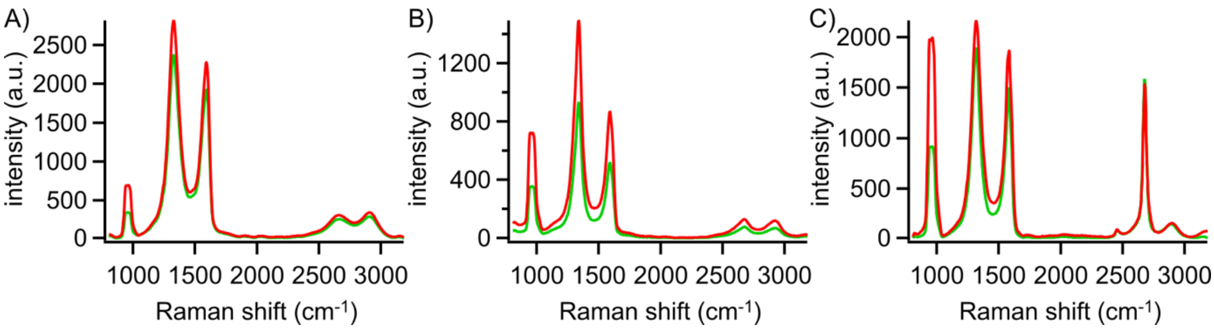

Fig. S5. Measured Raman spectra of the overlapped regions (green) in comparison to the sum of the Raman spectrum of each corresponding single layer in the heterostructures of A) oxo-G on oxo-G B) red-oxo-G on oxo-G and C) graphene on oxo-G.

AFM image of the heterostructure graphene/oxo-G/graphene

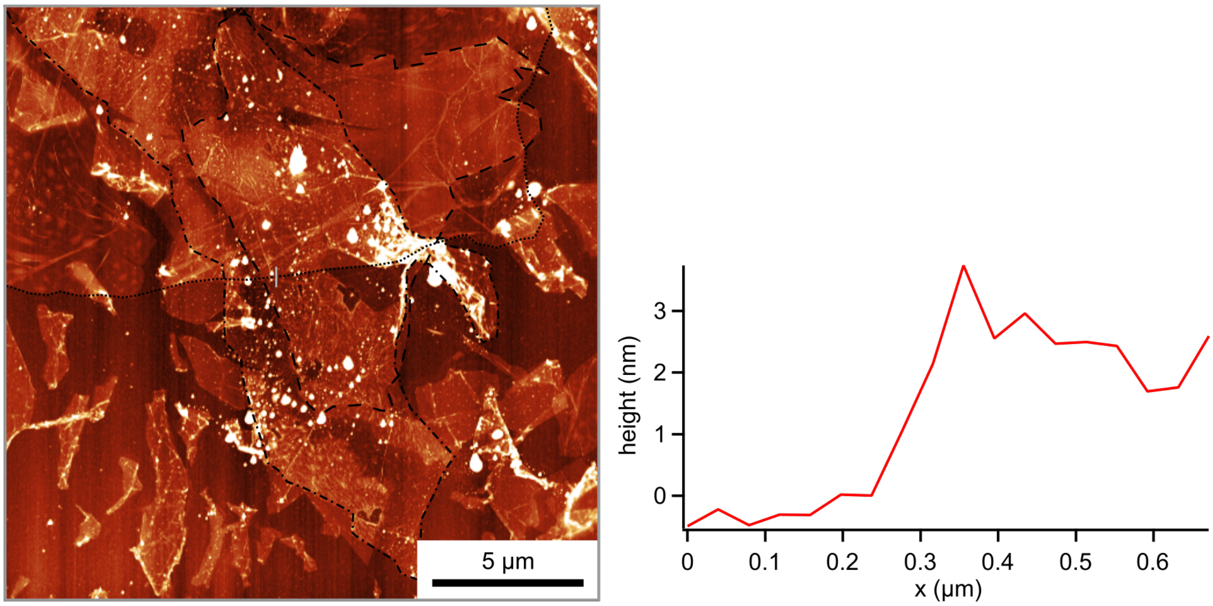

Fig. S6. AFM image of graphene/oxo-G/graphene with marked lines along 1st layer CVD graphene (dashed),2nd layer oxo-G (dotted) and 3rd layer CVD graphene (dashed and dotted), right: height profile along grey line.

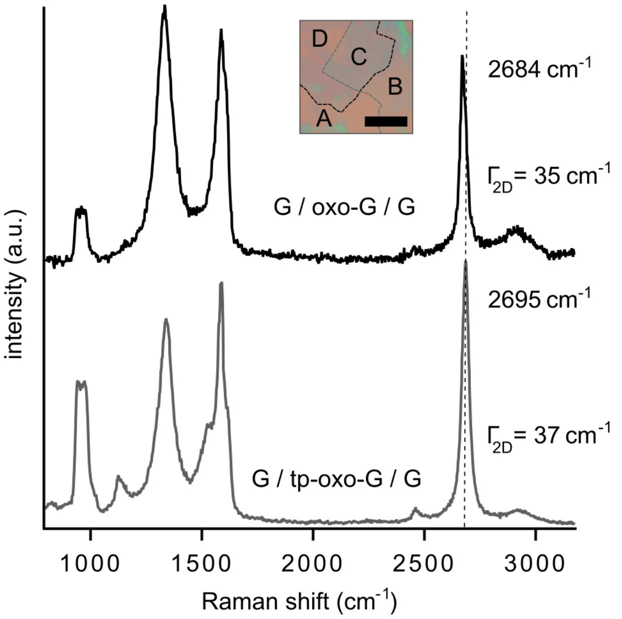

Fig. S7. Raman spectra of graphene/oxo-G/graphene (G/oxo-G/G, black line) and thermally processed (tp) for 1 h at 260 °C under an argon flow (G/tp-oxo-G/G, grey line), dashed line at 2D peak identifies Raman shift, which may occur due to p-doping of oxo-G,[9] inset: microscopic picture of the heterostructure (C) containing as 1st layer CVD graphene (A), 2nd layer oxo-G (B) and 3rd layer CVD graphene (D), scale bar corresponds to 5 µm.

REFERENCES

1. S. Eigler, M. Enzelberger-Heim, S. Grimm, P. Hofmann, W. Kroener, A. Geworski, C. Dotzer, M. Rockert, J. Xiao, C. Papp, O. Lytken, H. P. Steinrück, P. Müller and A. Hirsch, *Adv. Mater.*, 2013, **25**, 3583-3587.
2. K. B. Blodgett, *J. Am. Chem. Soc.*, 1935, **57**, 1007-1022.
3. S. Eigler, F. Hof, M. Enzelberger-Heim, S. Grimm, P. Müller and A. Hirsch, *J. Phys. Chem. C*, 2014, **118**, 7698-7704.
4. L. G. Cançado, A. Jorio, E. H. Ferreira, F. Stavale, C. A. Achete, R. B. Capaz, M. V. Moutinho, A. Lombardo, T. S. Kulmala and A. C. Ferrari, *Nano Lett.*, 2011, **11**, 3190-3196.
5. M. M. Lucchese, F. Stavale, E. H. M. Ferreira, C. Vilani, M. V. O. Moutinho, R. B. Capaz, C. A. Achete and A. Jorio, *Carbon*, 2010, **48**, 1592-1597.
6. P. J. Zomer, S. P. Dash, N. Tombros and B. J. van Wees, *Appl. Phys. Lett.*, 2011, **99**, 232104.
7. R. Frisenda, E. Navarro-Moratalla, P. Gant, D. Perez De Lara, P. Jarillo-Herrero, R. V. Gorbachev and A. Castellanos-Gomez, *Chem. Soc. Rev.*, 2018, **47**, 53-68.
8. X. Ma, Q. Liu, D. Xu, Y. Zhu, S. Kim, Y. Cui, L. Zhong and M. Liu, *Nano Lett.*, 2017, **17**, 6961-6967.
9. A. Das, S. Pisana, B. Chakraborty, S. Piscanec, S. K. Saha, U. V. Waghmare, K. S. Novoselov, H. R. Krishnamurthy, A. K. Geim, A. C. Ferrari and A. K. Sood, *Nature Nanotech.*, 2008, **3**, 210-215.
